# Impact of molar incisor hypomineralization on oral health–related quality of life in 8–10-year-old children

**DOI:** 10.1007/s00784-021-04150-w

**Published:** 2021-08-27

**Authors:** Taneeya Joshi, Alexander Rahman, Sabine Rienhoff, Jan Rienhoff, Tanja Stamm, Katrin Bekes

**Affiliations:** 1 Private Practice “Magic Dental”, Hunaeusstr. 6, 30177 Hannover, Germany; 2 Department of Conservative Dentistry, Periodontology and Preventive Dentistry, Hannover Medical School, Carl-Neuberg-Straße 1, 30625 Hannover, Germany; 3 Center for Medical Statistics, Informatics, and Intelligent Systems, Section for Outcomes Research, Medical University of Vienna, Spitalgasse 23, 1090 Vienna, Austria; 4 University Clinic of Dentistry, Department of Paediatric Dentistry, Medical University Vienna, Sensengasse 2a, 1090 Vienna, Austria

**Keywords:** Molar incisor hypomineralization, MIH, Child Perceptions Questionnaire, CPQ8-10, Oral health–related quality of life, OHRQoL

## Abstract

**Objectives:**

The aim of this study was to compare oral health–related quality of life (OHRQoL) in children with and without molar incisor hypomineralization (MIH) and to assess the impact of severity of MIH on OHRQoL in children between 8–10 years using the German version of the Child Perceptions Questionnaire (CPQ-G8-10).

**Materials and methods:**

Children aged 8–10 years were recruited at a pediatric dental clinic in Hannover, Germany. Half of them were affected by MIH. Participants were evaluated for presence and severity of MIH (MIH-TNI), plaque and dental caries status. Children were asked to answer the CPQ-G8-10. Statistical analysis was performed using GraphPad Prism-software version 8.

**Results:**

One hundred eighty-eight children (mean age 8.80 [± 0.84]; 43.10% female) were included in the study with 94 children having MIH. CPQ-G8-10 mean scores in MIH-affected children were significantly higher than in children showing no MIH (13.87 [± 8.91] vs. 4.20 [± 3.74]; *p* < 0.0001) showing that MIH has negative impact OHRQoL. Similar trends were seen in all four subdomains. Regarding severity, CPQ-G8-10 mean scores increased from mild to severe forms of MIH.

**Conclusion:**

Children affected by MIH show an impaired OHRQoL compared to children without MIH; with increasing severity, OHRQoL gets more impaired.

**Clinical relevance**

To understand the patient’s perception and the individual oral health needs will help to prioritize MIH and recognize its impact.

**Supplementary Information:**

The online version contains supplementary material available at 10.1007/s00784-021-04150-w.

## Introduction

Molar incisor hypomineralization (MIH) is a phenomenon defined as a quality defect of enamel of systemic origin of one to four permanent first molars frequently associated with affected incisors [1]. It affects around 13.1% of the global population that is around 878 million people. Out of these, 27.4% of the cases are affected with symptoms and post-eruptive enamel breakdown (PEB) and are in need of treatment [2]. In Germany, the average prevalence of MIH is 10.1%, with a variation from 4.3 to 14.6% in different regions [3]. Clinically, the lesions are porous and appear as well-demarcated opacity, mostly on the occlusal and buccal areas of the tooth surface which are usually more than 1 mm, and the color ranges from white, creamy or yellow, to brownish [4].

MIH-affected children face a number of difficulties in their daily life. Due to the porosity in severely affected enamel, masticatory forces break down the tooth surfaces, resulting in unprotective dentine in large atypical cavities. Exposed dentine causes hypersensitivity or rapid formation of caries [5]. These teeth are not only temperature-sensitive but can also cause pain on mechanical stimulus. This may adversely affect even the simplest of the daily vital activities like brushing or eating [6, 7]. In addition, aesthetics also plays an important role when the incisors are affected. Incisal defects are pretty extensive occurring mainly on the buccal surface leading to the aesthetic concern. The children’s appearance not only upsets the mothers but also has a negative influence on the children, preventing them from smiling [8]. Such aesthetics may lead to a negative social impact [7].

These problems described above may affect oral health–related quality of life (OHRQoL) in MIH patients. It is known that OHRQoL is an integral part of one’s well-being and general health [9]. The subjective evaluation of OHRQoL “reflects people’s comfort when eating, sleeping, and engaging in social interaction; their self-esteem; and their satisfaction with respect to their oral health” [10]. For children aged 8 to 10 years, the Child Perceptions Questionnaire (CPQ8-10) is the most frequently used instrument to measure OHRQoL in this age group [11]. It is a generic questionnaire which was designed to cover a variety of oral conditions.

Although MIH is known for 20 years now, there is still scarce data about the relationship between MIH and OHRQoL. Very few international studies have focused on this topic and have also shown varying results [12–14]. To date, only one study compared OHRQoL in children with and without MIH in Mexico [12]. However, these study patients were not matched but taken from a cross-sectional study. Furthermore, no study has focused on this topic in Germany yet.

Therefore, the aim of this study was to compare OHRQoL in children with and without MIH and to assess the impact of severity of MIH on OHRQoL in children between 8 and 10 years using the German version of the CPQ-G8-10.

## Materials and methods

### Design

For this prospective matched pairs study, German children aged 8 to 10 years were recruited at the “Magic Dental Clinic”, Hannover, Germany. The enrolment into this study was voluntary. Extended information leaflets on the aim of the study were handed out and explained to the parents who gave their written and oral informed consent. The approval for the study procedures was granted by the ethics committee of the local University Review Board (Hannover Medical School, 9158_BO_K_2020).

### Subjects and setting

All dentists of the dental clinic were briefed about the details of the study and asked to recruit patients. A calibrated examiner (TJ) performed all clinical examinations. The criteria proposed by the European Academy of Paediatric Dentistry (EAPD) [4] were used for the diagnosis of MIH. These include the presence of demarcated opacities, post-eruptive enamel breakdown, atypical restorations, and extraction due to MIH in at least one first permanent molar. Demarcated opacities with a diameter of < 1 mm were not considered in the analysis.

German-speaking children between 8 and 10 years of age and with fully erupted all first permanent molars were included in the study. Exclusion criteria were systemic comorbidities**,** physical or mental disability, severe malocclusion, or undergoing orthodontic treatment**,** enamel developmental defects other than MIH.

According to the sample size calculation, a total of 188 subjects were required for the present study. The formula that was used for sample size calculation was *n* = (*Z α*/2 + *Z β*) 2 * 2 * *σ*2/*d* 2, where *Z α*/2 was the critical value of the normal distribution at *α*/2 (confidence level is of 95%, and therefore, *α* was 0.05 and the critical value was 1.96), *Z β* was the critical value of the normal distribution at *β* (power of 80% is considered and therefore *β* was 0.2 and the critical value is 0.84), *σ*2 is the population variance, and *d* is the difference which is considered to be 10. Based on this calculation, a total of 157 subjects were needed. After adding a 20% drop out rate, 188 children were recruited. The subjects were evenly divided into two groups. Group I consisted of 94 subjects with MIH and group II consisted of 94 subjects without MIH.

### Data collection

During a single visit to the dental clinic, subjects were instructed to first brush their teeth followed by a clinical examination to grade the presence or absence of MIH, dental caries status using the dmft/DMFT index, and the plaque status using the Approximal Plaque Index (API). In case of presence of MIH, children were further examined for the severity of MIH using the MIH treatment need index (MIH-TNI) [15]. The MIH-TNI takes into account two most important clinical symptoms: post-eruptive breakdown and hypersensitivity: MIH-TNI 1 no breakdown, no hypersensitivity; MIH-TNI 2 breakdown, no hypersensitivity; MIH-TNI 3 no breakdown, hypersensitivity; MIH-TNI 4 breakdown, hypersensitivity.

To assess the child’s OHRQoL, the validated German version of the CPQ8-10 was used [16]. The CPQ8-10 contains a total of 25 items which can be subdivided into four domains: oral symptoms (five items), functional limitations (five items), emotional well-being (five items), and social well-being (ten items). Questions ask about the frequency of events in the child’s last 4 weeks. Responses are made on an ordinal scale (0 = ever, 1 = once/twice, 2 = sometimes, 3 = often, 4 = every day/almost every day). Higher scores refer to a worse OHRQoL status. Summing the response codes for the questionnaire items generates domain scores/sub-scales and an overall CPQ-G8-10 score. The instrument’s summary score ranges from 0 to 100. A summary score of zero indicates the absence of any problems, and higher CPQ scores represent more impaired OHRQoL. In addition to the 25 items, the CPQ-8–10 includes two questions asking the child for a global rating of the oral health and the overall well-being. These global ratings had a five-point response format (excellent, very good, good, moderate, poor).

### Data analysis

After data collection, statistical analysis was done using GraphPad Prism software version 8. The D’Agostino-Pearson normality test was used to determine whether the sets of scores/indices to be compared followed a Gaussian distribution or not. Based on this, either the unpaired Student *t*-test or the Mann–Whitney test was performed for comparison between two groups. Wherever a comparison between multiple groups was involved, either the one-way ANOVA (analysis of variance) or the Kruskal–Wallis test was used to determine the statistical significance. A *p*-value of less than 0.05 was considered significant.

## Results

### Study population

A total of 188 subjects aged 8–10 years (43.1% female) were involved in this study. They were equally divided into two groups with 94 subjects affected with MIH (50% female). The mean age was 8.80 years (± 0.84 years) (Table 1). The mean dmft/DMFT score for the MIH group was 5.04 (± 3.73) and 5.49 (± 3.84) for the control demonstrating a comparable experience. A significant higher API was observed in children suffering from MIH (50.53% (± 33.72) vs. 40.34% (± 33.07)) (*p* < 0.05, Mann–Whitney test).

**Table 1:** General characteristics of participants

Characteristics	All	Control	MIH
Gender (*N*, %)	188 (100%)	94 (100%)	94 (100%)
Male	107 (56.9%)	60 (63.8%)	47 (50%)
Female	81 (43.1%)	34 (36.2%)	47 (50%)
Mean age (years, SD)	8.80 (0.84)	8.74 (0.79)	8.87 (0.88)
Mean dmft/DMFT score (SD)	5.27 (3.79)	5.49 (3.84)	5.04 (3.73)
Mean API (%)	45.29 (± 33.72)	40.34 (± 33.07)	50.53 (± 33.72)

### Oral health–related quality of life in patients with and without MIH

The mean CPQ-G8-10 score for MIH-affected children was 13.87 (± 8.91; median 12; range 0–48) while the control group showed a reduced score of 4.20 (± 3.73; median 3; range 0–18), indicating a better self-perceived oral-health (Table 2, Supplement). The scores of the subdomains of the CPQ-G8-10 followed the same patterns. Oral symptoms (6.88 [± 3.76] vs. 2.29 [± 2.60]), functional limitations (2.35 [± 2.36] vs. 0.88 [± 1.35]), emotional well-being (2.91 [± 3.23] vs. 0.66 [± 1.24]), and social well-being (1.72 [± 2.62] vs 0.38 [± 0.96]) were significantly increased in MIH patients (*p* < 0.001, *t*-test). Furthermore, female MIH patients showed a higher total score compared to males (15.96 [± 9.99] vs. 11.79 [± 7.20]) which was statistically significant. As expected, MIH patients’ high scores were observed in the subdomain “oral symptoms” which includes questions on the frequency of pain.

**Table 2:** CPQ-G8-10 mean scores in patients with and without MIH

	Total score	Oral symptoms	Functional limitations	Emotional well-being	Social well-being
Control (MIH-TNI 0)					
All	4.20 (± 3.74)	2.29 (± 2.60)	0.88 (± 1.35)	0.66 (± 1.24)	0.38 (± 0.96)
Female	4.50 (± 3.69)	2.03 (± 2.12)	0.97 (± 1.45)	0.88 (± 1.61)	0.62 (± 1.81)
Male	4.03 (± 3.79)	2.43 (± 2.83)	0.82 (± 1.30)	0.53 (± 0.96)	0.25 (± 0.80)
MIH					
All	13.88 (± 8.91)	6.88 (± 3.76)	2.35 (± 2.36)	2.91 (± 3.23)	1.72 (± 2.62)
Female	15.96 (± 9.99)	7.51 (± 3.82)	2.55 (± 2.57)	3.68 (± 3.72)	2.21 (± 3.28)
Male	11.79 (± 7.20)	6.26 (± 3.62)	2.15 (± 2.14)	2.15 (± 2.45)	1.23 (± 1.63)
MIH-TNI 1 (*N* = 19)	7.26 (± 3. 94)	9.00 (± 2.31)	6.84 (± 1.83)	5.68 (± 1.00)	10.73 (± 0.93)
MIH-TNI 2 (*N* = 27)	9.63 (± 4.77)	10.44 (± 2.85)	6.74 (± 2.19)	6.74 (± 1.98)	10.70 (± 0.95)
MIH-TNI 3 (*N* = 18)	13.78 (± 6.73)	11.61 (± 3.35)	6.94 (± 1.96)	8.00 (± 2.05)	12.22 (± 2.41)
MIH-TNI 4 (*N* = 30)	21.93 (± 9.24)	15.17 (± 3.09)	8.47 (± 2.67)	10.33 (± 4.01)	12.97 (± 3.67)

### Oral health–related quality of life in MIH patients with regard to severity of the condition

**Fig. 1:**
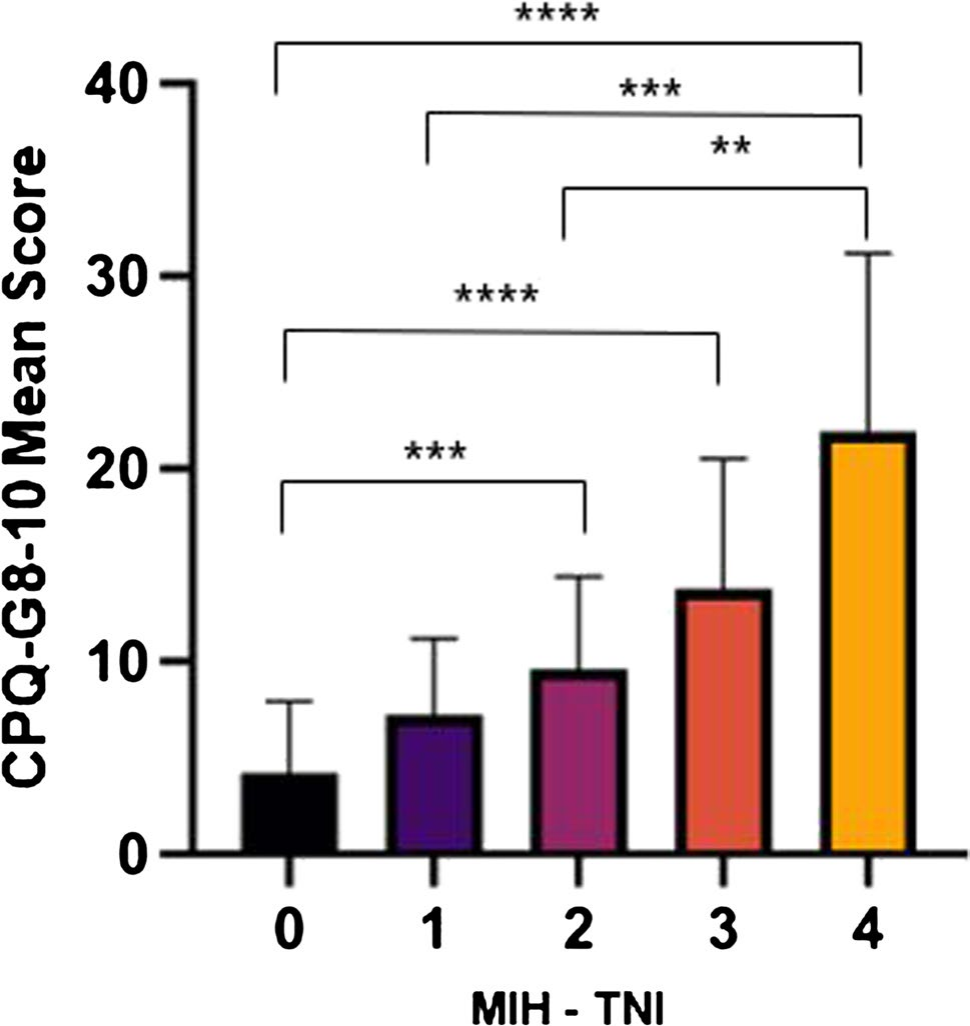
CPQ-G8-10 means in controls (MIH-TNI 0) and in MIH patients with different severities (MIH-TNI 1–4); **p < 0.01, ***p < 0.001, ****p < 0.0001

Within the MIH patient group, the mean CPQ-G8-10 score increased with the severity (Table 2, Fig. 1). In children with mild affected MIH teeth (MIH-TNI 1), a mean score of 7.26 (± 3.76) was observed increasing to 21.93 (± 21.93) in patients with severe MIH (MIH-TNI 4). Pairwise comparison showed statistically significant differences between the control group and MIH-TNI 2, MIH-TNI 3, and MIH-TNI 4 (*p* < 0.001, Kruskal–Wallis test). Although higher scores were already recorded in mild MIH cases (MIH-TNI1), this difference was not significant compared to the control group (*p* > 0.05, Kruskal–Wallis test).

### Most often reported problems

The five most often ticked items of MIH patients were “pain or sensitivity towards cold drinks and foods” (*N* = 72, 76.60%), “bad breath” (*N* = 81, 86.17%), “pain in the teeth or mouth” (*N* = 59, 62.77%), “needed longer time than others to eat meal because of teeth or mouth” (*N* = 40, 42.55%) and “been upset because of teeth or mouth” (*N* = 35, 37.23%). The item “pain in teeth or mouth” showed a sevenfold increase patients with MIH compared to the patients without MIH. There were also some problems which were seen only in the MIH group, but not in the control group. They were frustrated because of the teeth or mouth (25.53%) and also had a hard time attending school (11.70%)/doing homework (4.26%) or paying attention in school (5.32%). They also did not want to talk (3.19%) or be with other children (2.13%) because of the teeth or mouth and were teased (3.19%) by other children because of the teeth or mouth.

## Discussion

This study compared OHRQoL in German children with and without MIH presenting in a private pediatric dental practice and also assessed the impact of severity of MIH. Although some authors have already studied the effect of MIH on OHRQoL [7, 12–14, 17–19], this is the first study focusing on this topic in Europe, using a comparable sample size of healthy and affected children and taking into account the severity of the condition by using the MIH-TNI. The study shows that children suffering from MIH have impaired OHRQoL which is decreasing with higher severity of MIH.

In the present study, the CPQ 8–10 was used to measure OHRQoL. The instrument is frequently used worldwide and has already been translated and validated in several countries. In this study, the German version was applied [16]. The CPQ 8–10 was developed for children aged 8–10 years. Therefore, the focus was placed on this age range in the study. No younger patients were included as Weerheijm et al. recommended that 8 years of age should be considered the best time for any examination for the condition as at this age, in most children, all 4 permanent molars will be erupted, as will be the majority of the incisors, while signs of MIH will still be present [4].

In this study, the presence of MIH significantly impacted OHRQoL compared to the control group (13.88 [± 8.91] vs. 4.20 [± 3.74]). Children affected with MIH showed a 3 times more impaired OHRQoL.

Furthermore, regarding severity of MIH, mean CPQ-G8-10 scores increased with increasing severity showing a more impaired OHRQoL in severe cases. In children with mild affected MIH teeth (MIH-TNI 1), a mean score of 7.26 (± 3.76) was observed increasing to 21.93 (± 21.93) in patients with severe MIH (MIH-TNI 4) which was statistically significant. However, it should be noted that although higher scores were already recorded in mild MIH cases (MIH-TNI1), this difference was not significant compared to the control group.

Similar results were observed in some other studies. Velandia et al. [18] investigated OHRQoL applying the same questionnaire in 88 children aged 7–10 years with half of them showing MIH. The mean CPQ-8–10 score in MIH patients was 17.4 compared to 4.3 in healthy patients. They also assessed the impact of severity using the severity index by Mathu Muju and Wright [20], but failed to show any statistical significance. However, it should be mentioned that this index does not take into account the possible presence of hypersensitivity of an MIH tooth which might have an influence on OHRQoL. It only focuses in the presence of post-eruptive breakdowns. Furthermore, they only included a very small number of participants (less than 10%) with severe MIH. A further study done in Mexico using a sample of 411 schoolchildren (8–10 years old) confirmed the negative impact on MIH-affected children’s OHRQoL [12]. Children with moderate/severe MIH experienced a greater impact across the four domains compared to children without MIH. Similarly, Shojaeepour et al. [21] measured OHRQoL of 129 Iranian children between 8 and 12 years of age suffering from MIH. A mean score 19.9 was observed. Unfortunately, no control group was included in this study and that no comparison to our control group can be done.

Further studies focusing on OHRQoL in MIH using other approaches for measurement point in the same direction. In another study performed to evaluate the OHRQoL in MIH children, both, the parents and children, were separately asked to fill in the questionnaire. A negative impact on OHRQoL was also found; however, it should be noted that there was poor agreement between the children and their parents [8].

It is important to understand that the QoL is not only limited to the effects caused by MIH on the oral health. It is multidimensional and affects not only psychological aspects such as emotional and social well-being, but also physical symptoms and functioning [22]. Although a negative impact of MIH was seen in all four domains, namely oral symptoms, functional limitations, emotional well-being, and social well-being, we were able to show that the highest scores were observed in the oral symptoms domain. This is also in accordance with other studies also showing the highest impacts in this domain and observing similar scores [13, 18, 21]. Children with MIH were most frequently affected with pain.

Regarding gender, it was observed that MIH-affected female patients showed more impaired OHRQoL than male patients. Other studies have shown similar results [14, 18, 21]. It can be speculated that this may be due to the fact that female patients are more worried about their self-perception and their appearance [23].

There are some strengths and limitations of this study. One of the limitations is the study design. As this was a cross-sectional study, the present design does not measure the effect prospectively but at a certain point of time in the life of the participant. Furthermore, it is noteworthy that a special setting was used in our investigation which makes a direct comparison with other studies challenging due to differences in study designs. Almost all published studies are prevalence studies being conducted at school. The current investigation was performed in a private pediatric dental clinic. Even though some patients come for routine checkups, most show up due to the presence of some uncomfortable symptoms. Therefore, the patient population is highly selective which can be seen regarding the high dmft/DMFT of the control group. However, the results of this study show that children without MIH but with a comparable and dmft/DMFT already show a better OHRQoL than MIH-affected children. Since it is already known that caries-free children have an even better OHRQoL (lower CPQ scores) compared to children with caries [16], the differences between these children and MIH children would certainly be even more notable.

Despite all the limitation, this study offers relevant perspective on OHRQoL in MIH-affected children. One of the strengths of the study is that this was the first study evaluating the impact of severity on OHRQoL in MIH patients using the MIH-TNI. The MIH-TNI is a promising index as it takes into account the two most important clinical symptoms of MIH: post-eruptive breakdown and hypersensitivity. Other indices only focus on the breakdown. However, hypersensitivity plays an important role in clinical context. As a small limitation regarding the application the index, it has to be noted for this study that no subdivision into the categories of the MIH-TNI 2 and MIH-TNI 4 (size of the post-eruptive breakdown) was undertaken.

## Conclusion

Children with MIH have a poorer OHRQoL as compared to children without MIH when applying the CPQ-G8-10 questionnaire. The negative impact was seen in all the four domains, with maximum difference being observed in the oral symptoms domain. Children having severe (MIH-TNI 4) and moderate MIH (MIH-TNI 2 and MIH-TNI 3) had a poorer OHRQoL than those with mild (MIH-TNI 1) or no MIH.

## Supplementary Information

Below is the link to the electronic supplementary material.

Supplementary file1 (DOCX 23 KB)
