# Supplementary material for: Impact of molar incisor hypomineralization on oral health–related quality of life in 8–10-year-old children
Source: Clin Oral Investig. 2021 Aug 27;26(2):1753–9. doi: 10.1007/s00784-021-04150-w (PMC8816800; doi:10.1007/s00784-021-04150-w)
Supplement: Supplementary file 1 — Supplementary file1 (DOCX 23 KB) [file 784_2021_4150_MOESM1_ESM.docx]

**Supplementary table:** CPQ-G8-10 mean scores of all 25 items in patients with and without MIH

|  | Control (MIH-TNI 0) | MIH |
| --- | --- | --- |
| ***Total score*** | ***4.20 (±3.74)*** | ***13.88 (±8.91)*** |
| Pain in your teeth | 0.23 (±0.63) | 1.51 (±1.43) |
| Sore spots in your mouth | 0.17 (±0.63) | 0.40 (±0.80) |
| Pain in your teeth when you drink cold drinks or eat foods | 0.46 (±1.00) | 2.07 (±1.48) |
| Food stuck in your teeth | 0.47 (±1.03) | 0.84 (±1.16) |
| Bad breath | 0.96 (±1.14) | 2.05 (±1.20) |
| Needed longer time than others to eat your meal | 0.54 (±1.13) | 0.98 (±1.36) |
| Had a hard time biting or chewing food like apples, corn on the cob or steak | 0.18 (±0.49) | 0.78 (±1.11) |
| Had trouble eating foods you would like to eat | 0.02 (±0.21) | 0.30 (±0.84) |
| Had trouble saying some words | 0.10 (±0.44) | 0.04 (±0.25) |
| Had a problem sleeping at night | 0.03 (±0.18) | 0.26 (±0.70) |
| Been upset | 0.27 (±0.75) | 0.85 (±1.10) |
| Felt frustrated | 0 (±0) | 0.41 (±0.75) |
| Been shy | 0.16 (±0.53) | 0.45 (±0.91) |
| Been concerned | 0.07 (±0.40) | 0.53 (±0.97) |
| Worried | 0.16 (±0.51) | 0.67 (±1.07) |
| Missed school | 0.01 (±0.10) | 0.15 (±0.47) |
| Had a hard time doing your homework | 0 (±0) | 0.06 (±0.35) |
| Had a hard time paying attention in school | 0 (±0) | 0.07 (±0.34) |
| Not wanted to speak or read out loud in class | 0 (±0) | 0 (±0) |
| Tried not to smile or laugh when with other children | 0.13 (±0.55) | 0.52 (±1.00) |
| Not wanted to talk to other children | 0 (±0) | 0.06 (±0.44) |
| Not wanted to be with other children | 0 (±0) | 0.05 (±0.42) |
| Stayed away from activities like sports and clubs | 0 (±0) | 0.01 (±0.10) |
| Other children teased you or called you names | 0.01 (±0.10) | 0.09 (±0.52) |
| Other children asked you questions | 0.23 (±0.63) | 0.70 (±1.05) |
